# Mass Spectrometry of Putrescine, Spermidine, and Spermine Covalently Attached to *Francisella tularensis* Universal Stress Protein and Bovine Albumin

**DOI:** 10.1155/2024/7120208

**Published:** 2024-02-05

**Authors:** Lawrence M. Schopfer, Benjamin Girardo, Oksana Lockridge, Marilynn A. Larson

**Affiliations:** ^1^Eppley Institute, University of Nebraska Medical Center, Omaha, NE, USA; ^2^Pathology, Microbiology and Immunology Department, University of Nebraska Medical Center, Omaha, NE, USA

## Abstract

Bacterial and mammalian cells are rich in putrescine, spermidine, and spermine. Polyamines are required for optimum fitness, but the biological function of these small aliphatic compounds has only been partially revealed. Known functions of polyamines include interaction with nucleic acids that alters gene expression and with proteins that modulate activity. Although polyamines can be incorporated into proteins, very few naturally occurring polyaminated proteins have been identified, which is due in part to the difficulty in detecting these adducts. In the current study, bovine albumin and the recombinant universal stress protein from *Francisella tularensis* were used as models for mass spectrometry analysis of polyaminated proteins. The proteins were covalently bound to putrescine, spermidine, or spermine by the action of carbodiimide or microbial transglutaminase. Tryptic peptides, subjected to liquid chromatography tandem mass spectrometry (LC-MS/MS), were identified using Protein Prospector software. We describe the search parameters for identifying polyaminated peptides and show MS/MS spectra for adducts with putrescine, spermidine, and spermine. Manual evaluation led us to recognize signature ions for polyamine adducts on aspartate, glutamate, and glutamine, as well as neutral loss from putrescine, spermidine, and spermine during the fragmentation process. Mechanisms for the formation of signature ions and neutral loss are presented. Manual evaluation identified a false-positive adduct that had formed during trypsinolysis and resulted in peptide sequence rearrangement. Another false positive initially appeared to be a 71 kDa putrescine adduct on a cysteine residue. However, it was an acrylamide adduct on cysteine for a sample extracted from a polyacrylamide gel. The information presented in this report provides guidance and serves as a model for identifying naturally occurring polyaminated proteins.

## 1. Introduction

Polyamines are required for optimal growth in all living cells [1]. Most organisms synthesize these critical compounds de novo and/or acquire them from external sources. *Escherichia coli* contain 32.2 mM putrescine and 6.88 mM spermidine. About 50% of putrescine and 90% of spermidine exist as polyamine-RNA complexes [2]. Mammalian cells also contain millimolar concentrations of putrescine, spermidine, and spermine [3], and these polyamines have been shown to differentially modulate nucleic acid and protein functions reviewed in [4]. There is also evidence for naturally occurring polyaminated proteins in mammalian cells. The best known example is the eukaryotic translation initiation factor eIF5A that is posttranslationally modified to incorporate a spermidine onto Lys 50, followed by hydroxylation of the spermidine adduct to make the amino acid hypusine [5]. Hypusinated eIF5A is essential for the growth and survival of eukaryotic cells. A second example is the covalent attachment of putrescine or spermidine onto Gln 63 of RhoA, which leaves this GTPase in a constitutively active state [6]. Signals sent by activated RhoA result in actin cytoskeleton remodeling. Much less is known about polyamine adducts on bacterial proteins. One study reported that recombinant human histones expressed in *E. coli* are modified by putrescine, spermidine, and spermine via a covalent linkage to glutamine [7]. We have shown that recombinant *Francisella tularensis* universal stress protein (Usp) expressed in *E. coli* is polyaminated on glutamine [8].

The observation that *E. coli* bacteria have the capacity to polyaminate recombinant human and bacterial proteins in the absence of added factors suggests that polyaminated proteins may exist in many organisms. Polyaminated proteins may have important functions, but their function can only be determined after polyaminated proteins are identified. Thus, our goal is to establish a sensitive mass spectrometry protocol for identifying polyaminated peptides. In this report, we created polyaminated bovine albumin and polyaminated *F. tularensis* Usp to serve as models for interpreting mass spectrometry fragmentation spectra. We generated polyaminated proteins using two different methods that included chemical modification with carbodiimide and enzymatic modification with transglutaminase. The chemical approach yielded polyamine adducts on glutamic and aspartic acid, whereas transglutaminase treatment yielded polyamine adducts exclusively on glutamine. Neither of these approaches produces adducts on lysine.

In this report, we show MS/MS spectra of peptides modified by putrescine, spermidine, and spermine and provide search parameters for finding polyaminated peptides using the Protein Prospector search engine. We also show the mechanisms for the formation of signature ions for glutamate-putrescine and aspartate-putrescine, along with the masses of observed and theoretical signature ions for polyamine adducts on glutamate, aspartate, and glutamine. Signature ions are useful for confirming the identity of polyaminated peptides. We provide evidence for neutral loss of portions of putrescine, spermidine, and spermine adducts caused by collision-induced dissociation and show how to identify neutral loss. We provide guidance on how to evaluate MS/MS spectra and how to search for alternative interpretations.

## 2. Materials and Methods

### 2.1. Materials

Bovine serum albumin (BSA) was obtained from Cell Signaling catalog number 9998. Recombinant *F. tularensis* universal stress protein with a C-terminal histidine tag (rUsp/His_6_) was expressed in *E. coli* BL21 (DE3) and affinity purified by Ni-NTA chromatography, as previously described [8]. Other compounds and enzymes used included spermine tetrahydrochloride (Sigma, catalog number S2876), spermidine trihydrochloride (Sigma, catalog number S2501), putrescine dihydrochloride (Sigma, catalog number P5780), 1-ethyl-3-(3-dimethylaminopropyl) carbodiimide (ProteoChem, catalog number C1100), recombinant microbial transglutaminase from *Streptomyces mobaraensis* (Zedira, catalog number T001), and sequencing-grade modified trypsin (Promega, catalog number V5113).

### 2.2. Chemical or Enzymatic Conjugation of Polyamines to Proteins

**Figure 1:**
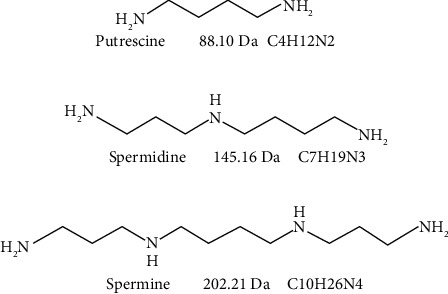
Structures of the polyamines putrescine, spermidine, and spermine. The monoisotopic molecular weights were calculated from their molecular formulas using Exact Mass Calculator, Single Isotope version from Scientific Instrument Services by Adaptus Solutions. Covalent binding of a polyamine to glutamine by transglutaminase causes the loss of NH_3_ (17 Da), whereas covalent binding of a polyamine to glutamate and aspartate via modification by 1-ethyl-3-(3-dimethylaminopropyl) carbodiimide causes the loss of H_2_O (18 Da).

Structures of the polyamines putrescine, spermidine, and spermine are shown in Figure 1.

The chemically generated polyaminated bovine serum albumin (BSA) was synthesized by treating 10 mg/mL BSA with 10 mg/mL putrescine, spermidine, or spermine in the presence of 2 mg/mL 1-ethyl-3-(3-dimethylaminopropyl) carbodiimide (EDC) in 0.1 M MES buffer (pH 6.5) containing 0.5 M sodium chloride at room temperature for 5 h. Excess reagents were removed by dialysis against 20 mM Tris-HCl (pH 8) containing 0.1% azide.

The enzymatically generated putrescine adduct on BSA was synthesized by incubating 2 mg/mL BSA in 0.5 mL of 20 mM imidazole, pH 7.5, 150 mM sodium chloride, 2 mM dithiothreitol, with 4 mM putrescine, and 0.066 mg/mL microbial transglutaminase for 2 h at 37°C. Transglutaminase labels glutamine residues with the polyamine, releasing ammonia (17 Da). Unlike mammalian transglutaminases, microbial transglutaminase does not require calcium ions for activity [9]. To inactivate transglutaminase, 0.5 M iodoacetamide was added to obtain a final concentration of 50 mM iodoacetamide. This step alkylated Cys 64, a component of the catalytic triad of microbial transglutaminase [9]. Excess putrescine was removed by dialysis against 20 mM Tris-HCl pH 8.

The enzymatically generated polyamine adducts on *F. tularensis* rUsp/His_6_ were synthesized by incubating 1 mg/mL rUsp/His_6_ with 1 mM spermine, spermidine, or putrescine in the presence of 10 *μ*g/mL microbial transglutaminase and 1 mM dithiothreitol in phosphate-buffered saline at pH 8.5 overnight at 37°C. The polyaminated rUsp/His_6_ protein was subjected to SDS PAGE and the band at 33 kDa was reduced, alkylated with iodoacetamide, and digested with trypsin.

### 2.3. Mass Spectral Analysis of Polyaminated Peptides

Polyamine-labeled protein preparations (0.2 mg) were reduced with 10 mM dithiothreitol, alkylated with 50 mM iodoacetamide, and digested with 4 *μ*g trypsin overnight at 37°C in 20 mM ammonium bicarbonate buffer (pH 8). Trypsin was inactivated by incubation in boiling water for 3 minutes. Peptides were bound to a Pierce C-18 spin column (Thermo Scientific, catalog number 89870), washed with 2% acetonitrile/0.1% trifluoroacetic acid, eluted with 40 *μ*L of 70% acetonitrile/0.4% trifluoroacetic acid, dried in a SpeedVac (Savant, model SC100), and dissolved in 100 *μ*L water. Peptide concentration was estimated to be 0.2 *μ*g/*μ*L from absorbance at 280 nm using a NanoDrop spectrophotometer (NanoDrop Technologies, model ND-1000). Approximately 5 *μ*L containing 1 *μ*g peptides was subjected to mass spectral analysis.

Liquid chromatography tandem mass spectrometry (LC-MS/MS) was performed as previously described [10]. Briefly, peptides were separated on a Thermo RSLC Ultimate 3000 ultra-high-pressure liquid chromatography system (Thermo Scientific) at 36°C. Solvent A was 0.1% formic acid in water and solvent B was 0.1% formic acid in 80% acetonitrile. Peptides were loaded onto an Acclaim PepMap 100 C-18 trap column (75 *μ*m × 2 cm; Thermo Scientific, catalog number 165535) at a flow rate of 4 *μ*L/minutes and washed with 98% solvent A/2% solvent B for 10 minutes. Desalted peptides were pumped onto a Thermo Easy-Spray PepMap RSLC C-18 column (75 *μ*m × 50 cm with 2 *μ*m particles, Thermo Scientific, catalog number ES803) and separated at a flow rate of 300 nL/minute using a gradient of 9–50% solvent B in 30 minutes, 50–99% solvent B in 40 minutes, held at 99% solvent B for 10 minutes, 99–9% solvent B in 4 minutes, and then held at 9% solvent B for 16 minutes.

Eluted peptides were sprayed directly into a Thermo Orbitrap Fusion Lumos Tribrid mass spectrometer (Thermo Scientific). Data were collected using data-dependent acquisition. A survey full-scan MS (from 350 to 1800 m/z) was acquired in the Orbitrap with a resolution of 120,000. The AGC target (Automatic Gain Control for setting the ion population in the Orbitrap before collecting the MS) was set at 4 × 10^5^ and the ion filling time was set at 50 msec. The 25 most intense ions with charge state of 2–6 were isolated in a 3 sec cycle and fragmented using high-energy collision-induced dissociation with 35% normalized collision energy. Fragment ions were detected in the Orbitrap with a mass resolution of 30,000 at 200 m/z. The AGC target for MS/MS was set at 5 × 10^4^ and dynamic exclusion was set at 30 sec with a 10 ppm mass window. Data were reported in *∗*.raw format. The *∗*.raw data files were converted to *∗*.mgf files using MSConvert (ProteoWizard Tools from SourceForge) for analysis by Protein Prospector.

### 2.4. Protein Prospector Search for Polyaminated Peptides

Candidates for polyaminated peptides were identified by Protein Prospector v 6.4.9 (prospector.ucsf.edu/prospector/mshome.htm) database searches using Batch-Tag Web. The search parameters for Batch-Tag Web were as follows: Database was User protein. User Protein Sequence was the FASTA file for either BSA (NCBI accession number CAA76847) or *Francisella tularensis* SCHU S4 (NCBI accession number WP_003021757). Taxonomy was either Bos taurus or *Francisella tularensis*. Precursor charge range was 2, 3, 4, and 5. Parent Tol was 20 ppm, Frag Tol: 30 ppm. Instrument was ESI-Q-high-res. Digest was either trypsin or no enzyme. The no enzyme option is justified by the overnight tryptic digestion which provides ample opportunity for nonspecific cleavage. Max. missed cleavages were 2. Constant mods were none. Expectation Calc method was none. Variable mods were carbamidomethyl (C) and oxidation (M). User defined variable modifications were Putrescine (Q); C4H9N; mass modification range 70.9–71.3. Spermidine (Q); C7H16N2; mass modification range 127.9–128.4. Spermine (Q); C10H23N3; mass modification range 185.0–185.4. Putrescine (E); C4H8N; mass modification range 69.0–71.0. Spermidine (E); C7H15N2; mass modification range 126.0–128.0. Spermine (E); C10H22N3; mass modification range 184.0–186.0. Putrescine (D); C4H8N; mass modification range 69.0–71.0. Spermidine (D); C7H15N2; mass modification range 126.0–128.0. Spermine (D); C10H22N3; mass modification range 184.0–186.0.

Candidates for polyaminated peptides chosen by Protein Prospector were checked by manual evaluation. An acceptable polyaminated peptide had sequence intervals for essentially all of the amino acids in the sequence, plus the interval for the labeled amino acid. Exceptions were allowed for the first two residues in a b-ion series, which are typically unresolved, and for occasional unresolved amino acid pairs. Examples of acceptable MS/MS spectra are shown.

Caution must be exercised when the labeled residue is near a terminus of the peptide. Some amino acids and modified amino acids have the same mass as the polyamine modifications. For example, alanine and valine minus CO have masses of 71 Da, which could be mistaken for putrescine on glutamine (Q); serine minus amine has a mass of 70 Da, which could be mistaken for putrescine on glutamate (E) or aspartate (D); lysine, glutamine, and arginine minus CO have masses of 128 Da, which could be mistaken for spermidine. If these residues follow or precede the given peptide sequence, they could compromise the interpretation.

## 3. Results

### 3.1. Polyamine Adducts

**Figure 2:**
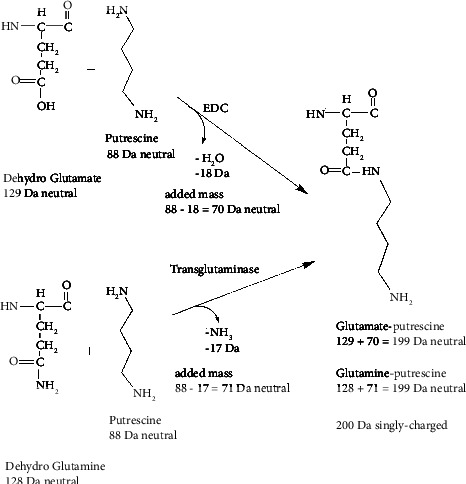
Formation of a glutamate-putrescine adduct catalyzed by EDC and formation of a glutamine-putrescine adduct catalyzed by transglutaminase. EDC catalyzes the covalent attachment of putrescine to the acid side chains of glutamic (E) or aspartic acid (D) to make an isopeptide bond. Transglutaminase catalyzes the covalent attachment of putrescine to the amide side chain of glutamine (Q) to make an isopeptide bond.

Products of the polyamine glutamate reaction catalyzed by EDC are the same as the products of the polyamine glutamine reaction catalyzed by transglutaminase. For example, Figure 2 illustrates the formation of the putrescine adduct on both glutamate and glutamine. The final, neutral mass of the adduct is 199 Da in both cases. In the case of glutamate reacting with putrescine catalyzed by EDC, the 199 Da mass is equal to the dehydro mass of glutamate plus the mass of putrescine minus water (129 + 88−18 Da). The added mass of putrescine is 70 Da. In the case of glutamine reacting with putrescine catalyzed by transglutaminase, the 199 Da mass is equal to the dehydro mass of glutamine plus the mass of putrescine minus ammonia (128 + 88−17 Da). The added mass of putrescine is 71 Da. When searching for adducts, we adjust the search parameters to recognize this one Dalton mass difference in added mass. Dehydro glutamate is glutamic acid as found in a peptide sequence. In an MS/MS spectrum, the intervals between amino acids are the dehydro masses. Analogous mechanisms can be drawn for spermine-glutamate/spermine-glutamine and spermidine-glutamate/spermidine-glutamine.  Table 1 shows a summary of the adduct masses.

The isopeptide bond is resistant to trypsin, which means the polyamine remains bound to a peptide during tryptic digestion. Trypsin-digested proteins subjected to LC-MS/MS allow identification of the modified protein, the modified residue, and the polyamine adduct.

**Figure 3:**
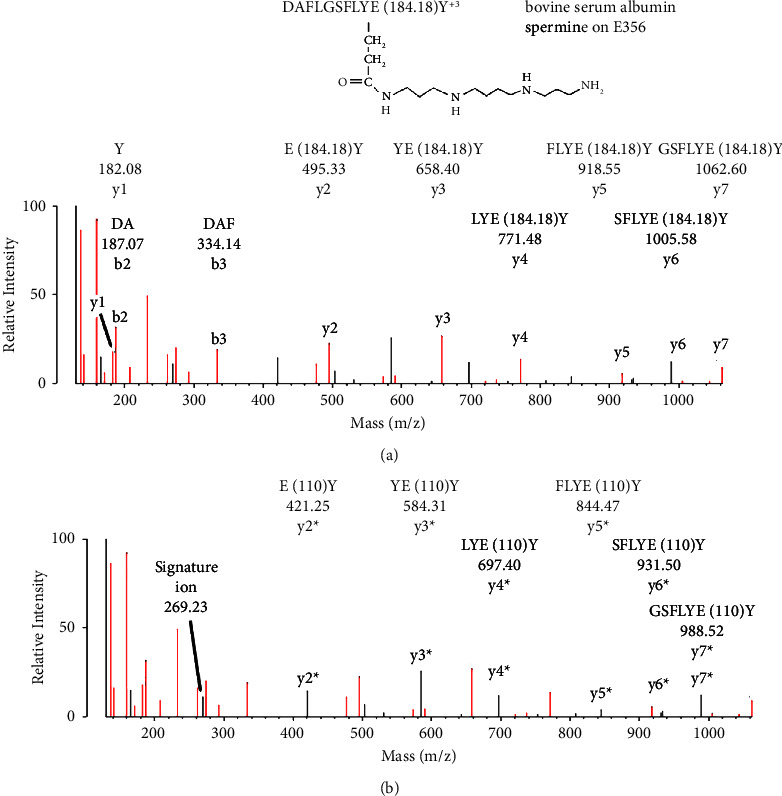
MS/MS spectrum for peptide DAFLGSFLYE(184.18)Y with spermine covalently bound to Glu 356 (E356) of bovine serum albumin, in a reaction catalyzed by EDC. The parent ion mass in charge state +3 is 503.61 m/z. (a) A y1–y7 ion sequence for the peptide. The mass difference between y1 and y2 ions is 313.25 Da, consistent with glutamate-spermine. There is also a short b-ion sequence, DAF, which completes the identification of all residues in the peptide except for b4, leucine. (b) The major unassigned peaks in the spectrum define a y-ion sequence that has undergone a neutral loss of 74 Da from spermine, leaving an added mass of 110 Da. The labeled fragments, y2^*∗*^–y7^*∗*^, are marked with an asterisk to denote the neutral loss. The mass of y2^*∗*^ (421.25) is consistent with glutamate (148.06 for the C-terminus of a y-series) plus tyrosine (163.06) plus 110 Da. This neutral loss was not anticipated, so the search engine was not configured to identify these fragments. Their presence was only revealed by manual evaluation. A nonsequence fragment at 269.23 Da is consistent with a signature ion that is unique for the glutamate-spermine construct. Most of the remaining, unassigned fragments correspond to internal fragments or to loss of amine, water, or CO from assigned fragments.

The mass difference between fragment ions in an MS/MS spectrum is a neutral mass. For example, the observed mass difference between y1 (182.08) and y2 (495.33) in Figure 3 is 313.25, a value that corresponds to the theoretical 313.22 neutral mass for the spermine adduct on glutamate, which is catalyzed by EDC and shown in Table 1. The fragment ions y1^+1^ (182.08) and y2^+1^ (495.33) and the parent ion are positively charged. In Figure 3, the parent ion is in charge state +3. Parent ions are generally in charge states +2, +3, or higher, while fragment ions are most often in charge states +1 or +2.

A total of eight mass spectral datasets were collected: four from EDC polyaminated BSA, one from transglutaminase polyaminated BSA, and three from transglutaminase polyaminated rUsp/His_6_. A total of 114,063 MS/MS spectra were collected. Protein Prospector identified 6474 polyaminated peptide candidates. These were screened for the presence of the correct added mass. Duplicates were eliminated, leaving only the candidate with the highest peptide score. This yielded 523 candidates that were manually evaluated. Application of our stringent criteria to 523 candidates resulted in 113 convincing polyaminated peptides. Accepted polyaminated peptides showed discriminant scores between 3.54 and 11.6, where a score greater than 0.0 is considered to be strong evidence for a correct match of the peptide sequence to the MS/MS spectrum. The discriminant score is calculated from the “best peptide score” and the “score difference” using the expression: discriminant score = −2.852 + (0.105 × best peptide score) + (0.11 × score difference). Where “best peptide score” is the score of the highest scoring peptide matched to a given protein, the score being calculated using a system of different values for different matching ion types. And, the “score difference” is the difference in score between the highest-scoring peptide and the second highest-scoring peptide [11].

### 3.2. Examples of MS/MS Fragmentation Spectra from Polyamine Labeled Peptides Produced by the Action of EDC on Bovine Albumin

Figure 3(a) shows an MS/MS spectrum for a BSA peptide that is labeled on Glu 356 by spermine, which was accomplished with EDC. The initial assignment was made by searching the data against the BSA user database with the search engine Protein Prospector and “No Enzyme” option. Protein Prospector assigned the peptide to DAFLGSFLYE(184.18)Y, in charge state +3 with a parent ion mass of 503.61 m/z. Spermine added 184.18 Da to Glu 356, which was confirmed by manual evaluation.  Figure 3(b) provides additional support for the spermine adduct on E356 of BSA. Manual evaluation showed that the y-ion series marked with an asterisk represents the spermine adduct on E356, where spermine has lost 74 Da by fragmentation in the mass spectrometer.

Our manual evaluation protocol considered alternative interpretations for the observed mass peaks. An added mass of 184 Da could originate from Ala (71) + Leu/Ile (113) or Ser (87) + Pro (97). The residues after the C-terminal in the BSA peptide are Ser and Arg (87 + 156), which cannot be mistaken for the added mass of 184 Da. In addition, the mass difference between the y1 and y2-ions supports the glutamate-spermine assignment.

In Table 1, the mass interval for spermine covalently bound to glutamate or glutamine is nearly identical at 313.22 and 313.24. The identity of glutamate as the modified residue relies on the known protein sequence of BSA (accession number CAA76847) and the fact that adducts formation was catalyzed by EDC.

**Figure 4:**
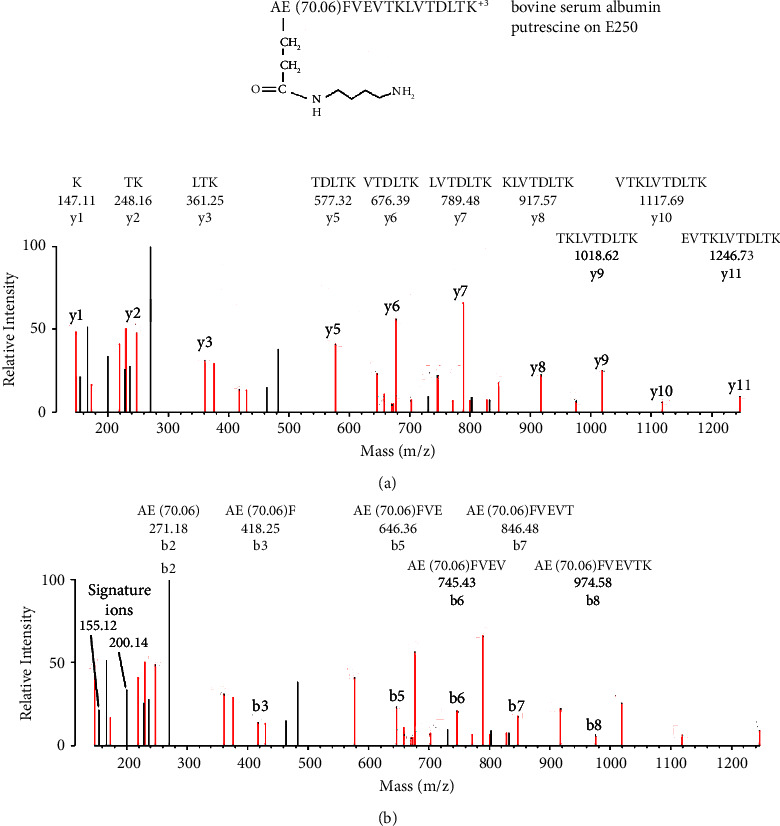
MS/MS spectrum for peptide AE(70.06)FVEVTLKLVTDLTK with putrescine covalently bound to Glu 250 of bovine serum albumin through the action of EDC. The parent ion mass in charge state +3 is 588.35 m/z. (a) A y-ion sequence, y1–y11, which identifies the peptide. This portion of the peptide does not include the labeled glutamate. (b) A b-ion sequence, b2–b8. All fragments in this sequence contain the added mass of 70.06 Da for the presence of putrescine. The mass of the b2 fragment (271.18) is consistent with alanine-glutamate-putrescine. The y-ion and b-ion sequences account for all residues in the peptide. The residue preceding the N-terminal side in the albumin sequence is lysine (dehydro mass 128.09), which cannot be mistaken for the added mass of 70 Da. Two nonsequence fragments at 155.12 and 200.14 Da are consistent with signature ions that are unique for the glutamate-putrescine construct. Most of the remaining, unassigned fragments correspond to internal fragments or to loss of amine, water, or CO from assigned fragments. Schemes that illustrate the formation of the signature ions are shown in Section 3.7.

Figure 4 shows an MS/MS spectrum for a peptide from BSA that is labeled on Glu 250 by putrescine. Labeling of glutamate (E250) was accomplished with EDC. The initial assignment was made by searching the data against the BSA user database with the search engine Protein Prospector and “No Enzyme” option. Protein Prospector assigned the peptide to AE(70.10)FVEVTLKLVTDLTK in charge state +3 with a parent ion mass of 588.35 m/z. Putrescine added 70.10 Da to Glu 250, which was confirmed by manual evaluation.

**Figure 5:**
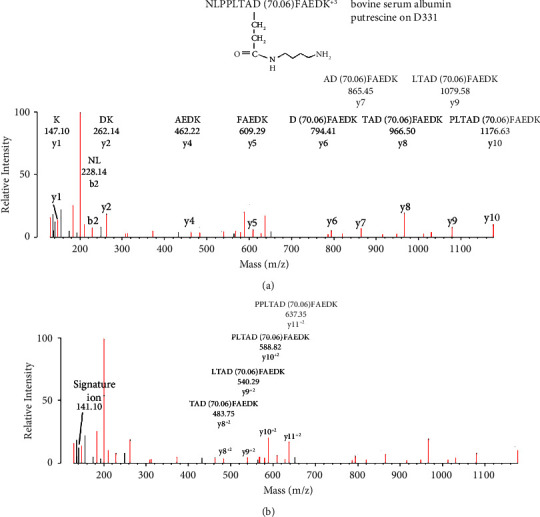
MS/MS spectrum for peptide NLPPLTAD(70.06)FAEDK^+3^ of bovine serum albumin covalently bound to putrescine at Asp 331 through the action of EDC. The parent ion mass in charge state +3 is 500.94 m/z. (a) A y-ion sequence, y1–y10. The interval between fragment y5 and y6 is consistent with aspartate-putrescine. Masses of the y7–y10 fragments are consistent with the presence of putrescine. The mass at 228.14 Da is consistent with the b2 ion, NL. (b) A doubly charged y-ion sequence for y8–y11. This sequence together with the sequences in panel (a) accounts for all of the residues in the peptide. A nonsequence fragment at 141.10 Da is consistent with a signature ion for the aspartate-putrescine construct. Most of the remaining, unassigned fragments correspond to internal fragments or to loss of amine, water, or CO from assigned fragments.

Figure 5 shows an MS/MS spectrum for a peptide from BSA that is labeled on Asp 331 by putrescine. Labeling of aspartate was accomplished with EDC, which couples carboxylate moieties to amines. The initial assignment was made by searching the data against the BSA user database with the search engine Protein Prospector and “No Enzyme” option. Protein Prospector assigned the peptide to NLPPLTAD(70.06)FAEDK, in charge state +3 with a parent ion mass of 500.94 m/z. Putrescine added 70.06 Da to Asp 331, which was confirmed by manual evaluation.

**Figure 6:**
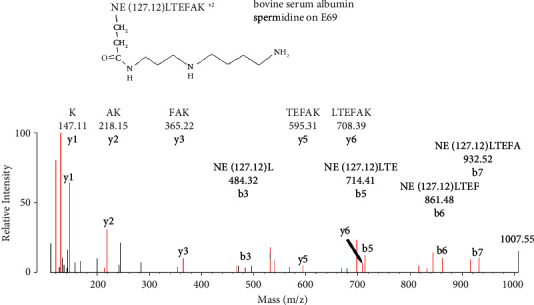
MS/MS spectrum for peptide NE(127.12)LTEFAK of bovine serum albumin covalently modified by spermidine on Glu 69 (E69) by the action of EDC. The parent ion mass in charge state +2 is 540.11 m/z. A y-ion sequence y1–y6 is present. This portion of the sequence does not include the labeled glutamate. A b-ion sequence, b3–b7, also is present. All of the masses in this sequence are consistent with the presence of spermidine added mass (127.12 Da). The y-ion and b-ion sequences account for all residues in the peptide. A nonsequence mass at 1007.55 Da is consistent with a neutral loss of 71 Da from spermidine on the parent ion. Most unlabeled fragments correspond to internal fragments or to loss of amine, water, or CO from assigned fragments.

Figure 6 shows an MS/MS spectrum for a peptide from bovine serum albumin labeled on Glu 69 by spermidine. Labeling of glutamate was mediated by EDC. The initial assignment was made by searching the data against the BSA user database with the search engine Protein Prospector and “No Enzyme” option. Protein Prospector assigned the peptide to NE(127.12)LTEFAK, in charge state +2 with a parent ion mass of 540.11 m/z. Spermidine added 127.12 Da to Glu 69, which was confirmed by manual evaluation.

### 3.3. Additional Polyaminated BSA Peptides Produced by the Action of EDC

Additional polyaminated peptides for bovine serum albumin produced by the action of EDC are listed in Table 2. Several forms of a modified peptide were found. For example, in Table 2, tryptic peptide LVNE^*∗*^LTEFAK modified on Glu 69 by putrescine was also found as peptides NE^*∗*^LTEFAK and VNE^*∗*^LTEFAK. Adducts were prepared separately for each polyamine, so that Glu 69 was found as a spermidine adduct as well as a putrescine adduct.

Signature ions were detected for some of the peptides. Signature ions are specific for the polyamine. For example, positively charged signature ions for putrescine adducts on glutamate and glutamine were observed at 129, 155, and 200 Da. Signature ions for spermidine adducts on glutamate and glutamine were observed at 186 and 212 Da. Signature ions for spermine adducts on glutamate were observed at 195 and 269 Da.

Neutral loss fragmentation was also detected for some of the peptides. Neutral loss constitutes the loss of a portion of the amine adduct from the parent ion. This type of fragmentation can result in a parallel sequence for which all the peptides are smaller than the normal sequence by the neutral loss mass, as shown in Figure 3. Peptides that are the consequence of neutral loss can exhibit signature ions that are different from the signature ions from the primary sequence.

Putrescine adducts were more abundant than spermidine and spermine adducts on BSA for reactions catalyzed by EDC. Table 2 lists 51 entries for putrescine adducts, 13 entries for spermidine adducts, and 13 entries for spermine adducts on glutamate and aspartate of BSA.

### 3.4. Example MS/MS Fragmentation Spectrum from Polyamine-Labeled Peptide Produced by the Action of Transglutaminase

**Figure 7:**
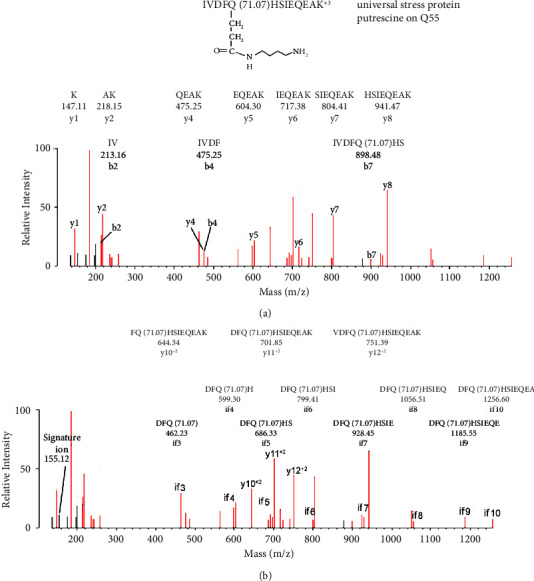
MS/MS spectrum for peptide IVDFQ(71.07)HSIEQEAK of rUsp/His_6_ covalently modified by putrescine on Gln 55 by the action of microbial transglutaminase. The parent ion in charge state +3 is 539.95 m/z. (a) A y-ion sequence, y1–y8, and a b-ion sequence, b2–b7. Only the b7 ion includes the putrescine adduct. The y4 and b4 masses are identical. (b) An internal fragment sequence from if3 to if10. Masses for all of the fragments in this series are consistent with the presence of the putrescine added mass (71.07 Da). In addition, fragments from a doubly charged sequence, y10^+2^ to y12^+2^, are consistent with the presence of the added mass for putrescine. The sequences in panels (a and b) account for all residues in the peptide. A nonsequence fragment at 155.12 Da is consistent with a signature ion for the glutamine-putrescine construct. Most unassigned fragments correspond to internal fragments or to loss of amine, water, or CO from assigned fragments.

Figure 7 shows an MS/MS spectrum for a peptide from rUsp/His_6_ covalently labeled on Gln 55 by putrescine, which was mediated by microbial transglutaminase. The initial assignment was made by searching the data against a Usp user database (accession number WP_003021757) with the search engine Protein Prospector and “No Enzyme” option. Protein Prospector assigned the peptide to IVDFQ(71.07)HSIEQEAK, in charge state +3 with a parent ion mass of 539.95 m/z. Putrescine added 71.07 Da to Gln 55, which was confirmed by manual evaluation.

### 3.5. Polyaminated Peptides Produced by the Action of Transglutaminase

Purified rUsp/His_6_ was incubated with putrescine, spermidine, or spermine in the presence of microbial transglutaminase. Tryptic peptides were subjected to LC-MS/MS, analyzed with Protein Prospector software, and confirmed by manual evaluation.  Table 3 lists the modified peptides and the signature ions. Neutral loss was not detected in these MS/MS spectra.

Reactions catalyzed by microbial transglutaminase yielded more putrescine adducts than spermidine and spermine adducts.  Table 3 lists 6 entries for putrescine adducts, 5 entries for spermidine adducts, and 3 entries for spermine adducts on rUsp/His_6_. As expected, only glutamine residues were modified by transglutaminase. Three of the 4 glutamine residues in rUsp/His_6_ are susceptible to transglutaminase-catalyzed polyamination.

Comparison of Tables 2–4 shows that putrescine signature ions on glutamate and glutamine have the same masses regardless of the catalyst that produced the adducts and regardless of the protein that was covalently modified by putrescine. The catalyst in Table 2 was the chemical EDC, while the catalyst in Tables 3 and 4 was transglutaminase. The protein in Tables 2 and 4 was BSA, while the protein in Table 3 was rUsp/His_6_. In contrast, putrescine adducts on aspartate have a different set of signature ions.

### 3.6. Putrescine Adduct on Cysteine Is Actually an Acrylamide Adduct on Cysteine

Our search for transglutaminase-mediated, polyaminated peptides in rUsp/His_6_ yielded an excellent candidate for a putrescine adduct on cysteine. 100% of the peaks in the MS/MS spectrum belonged to peptide VEHEVLVGNPAAEIVEYAEESNC(71.04)DVIVLGSHATH, in charge state +4 with a parent ion mass of 997.23 m/z. The added mass of 71.04 was consistent with putrescine added by transglutaminase. However, there is no precedent for transglutaminase labeling of cysteine with amines. Alternatively, a reaction between cysteine and acrylamide can occur if unalkylated proteins are subjected to SDS polyacrylamide gel electrophoresis. The product of the reaction is a propionamide adduct, which adds a mass of 71.04 Da [12]. The MS/MS fragmentation spectrum does not provide a means for differentiating the two possibilities. As such, this adduct could be mistaken for a putrescine adduct mediated by transglutaminase.

**Figure 8:**
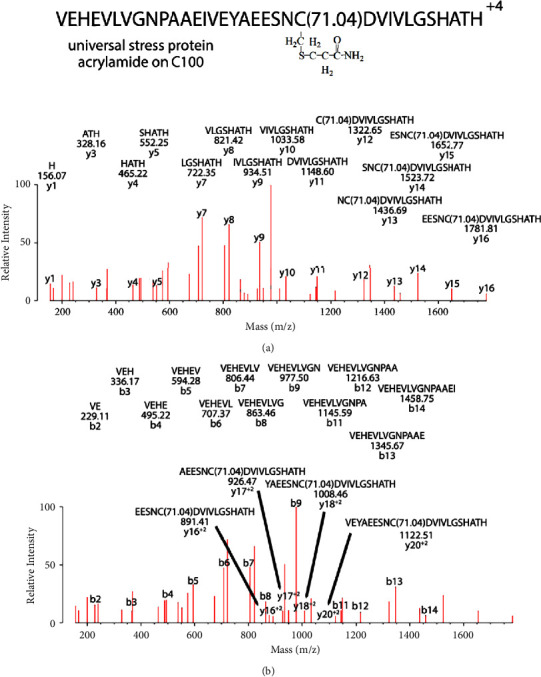
False positive putrescine adduct on cysteine is actually acrylamide adduct on cysteine. The MS/MS spectrum for VEHEVLVGNPAAEIVEYAEESNC(71.04)DVIVLGSHATH from rUsp/His_6_ shows Cys 100 covalently modified by acrylamide. The modification occurred spontaneously during polyacrylamide gel electrophoresis. The parent ion mass in charge state +4 is 997.23 m/z. (a) A y-ion sequence y1–y16. The interval between y11 and y12 is equal to the mass of cysteine (103 Da) plus the mass of propionamide (71.04 Da). The masses of peptides y12–y16 all are consistent with the presence of an added 71.04 Da. (b) The b-ion sequence b2–b14. None of these peptides includes the 71.04 Da added mass. There is also a doubly charged y-ion sequence y16–y20. The masses of these doubly charged peptides are consistent with the added mass of 71.04 Da. The y-ion, b-ion, and doubly charged y-ion sequences account for all of the residues in the peptide. Unassigned fragments correspond to internal fragments or to loss of amine, water, or CO from assigned fragments. A scheme showing addition of acrylamide to cysteine is in Figure 9.

Figure 8 shows the MS/MS spectrum of the peptide VEHEVLVGNPAAEIVEYAEESNC(71.04)DVIVLGSHATH labeled on Cys 100 of rUsp/His_6_, which added a mass of 71.04 Da. Preparation of the peptide involved incubation of partially purified rUsp/His_6_ with putrescine in the presence of microbial transglutaminase, followed by separation of the unalkylated mixture on SDS PAGE. Exposure of the preparation to unpolymerized acrylamide in the polyacrylamide gel makes acrylamide the most likely source of the 71.04 Da added mass.

**Figure 9:**
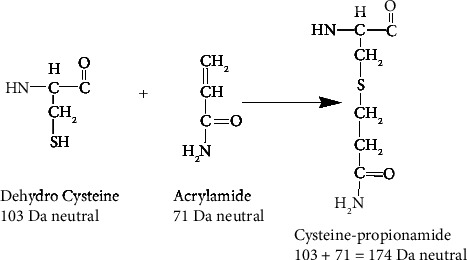
Scheme showing the addition of acrylamide to cysteine to form cysteine-propionamide with an added mass of 71 Da from acrylamide. The added mass from putrescine is also 71 Da. Acrylamide adducts on cysteine have been previously reported in proteins extracted from a polyacrylamide gel [12, 13].

Figure 9 shows a scheme that is consistent with the formation of cysteine-propionamide via a Michael addition [13]. The cysteine thiol attacks the *β*-carbon of *α*, *β*-unsaturated carbonyl with the assistance of a base. A proton from the cysteine-SH is lost, and a proton is added to the acrylamide. The net added mass is 71 Da.

### 3.7. Signature Ions from Polyaminated Adducts

Signature ions are low-mass, nonsequence fragments found in the MS/MS spectra of modified peptides. In the current context, they reflect the presence of the polyamine-modified amino acids. As such, they reinforce the identification of polyaminated peptides. Signature ions do not appear in all MS/MS spectra, and not all theoretical signature ions are observed.  Table 5 lists the observed (bold) and theoretical (black) signature ions for the polyamine adducts in this study. The first line in each section of Table 5 is for fragments that derive directly from the polyamine adducts, while the second line is for fragments that derive from polyamine adducts that have undergone neutral loss. Signature ions are independent of the protein. Analysis of the MS/MS spectra identifies the peptide, the polyaminated protein, the polyaminated residue, and the type of polyamine.

**Figure 10:**
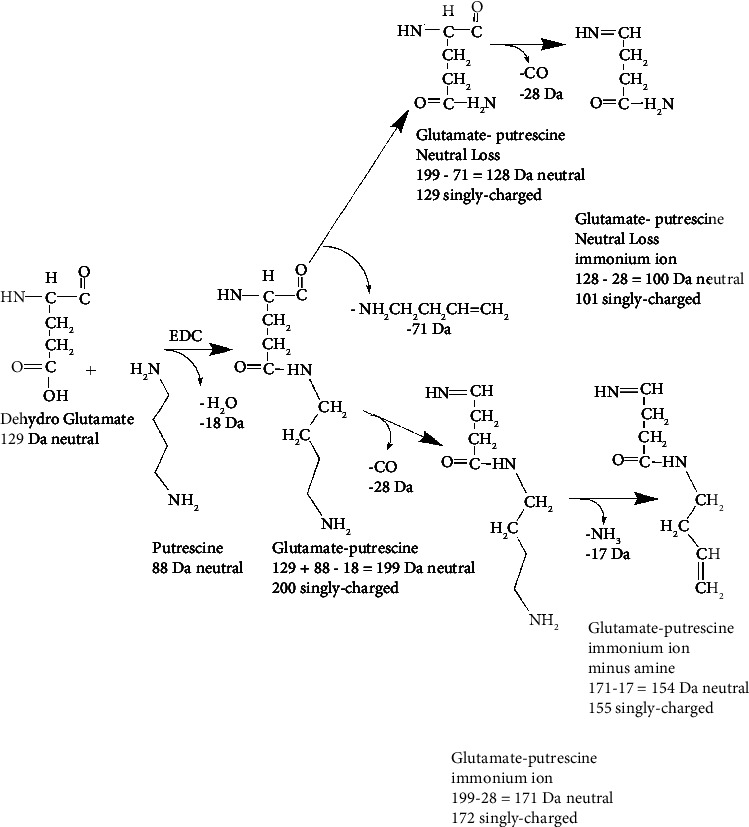
A scheme showing the structures of neutral loss fragments (upper branch) and signature ions (upper and lower branches) from the reaction of glutamate and putrescine. The neutral loss of 71 Da yields an observed signature ion at 129 Da, while the initial reaction yields the observed signature ion at 200 Da. Loss of CO and amine from the 200 Da adduct creates the observed signature ion at 155 Da.

Most of the observed signature ions are from either the adduct formed by the dehydro residue covalently attached to the polyamine or from the ion formed by loss of CO and amine from the initial adduct. For example, the reaction of dehydro glutamate with putrescine creates the singly charged 200 Da signature ion shown in the lower pathway of Figure 10. This ion was observed eight times. A second signature ion at 155 Da was created by loss of CO and NH_3_ from the 200 Da adduct. This ion was observed ten times. The theoretical, singly charged ion at 172 Da, created by the loss of CO (−28), was not observed. Similar mechanisms can be drawn for the formation of signature ions from other reaction products.

**Figure 11:**
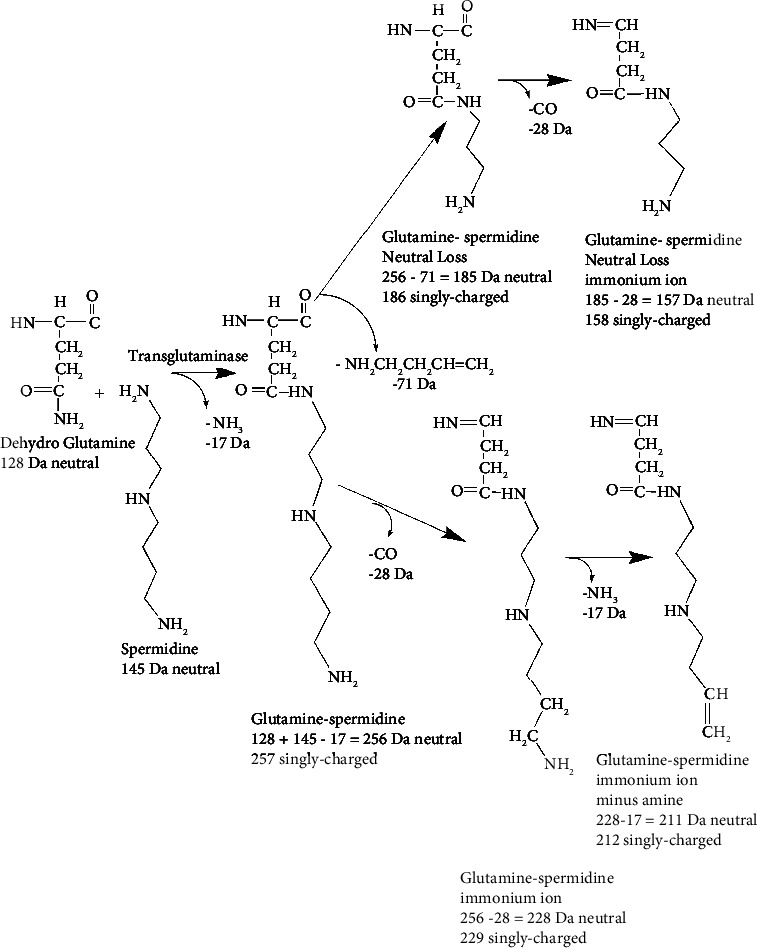
A scheme showing the structures of neutral loss fragments (upper branch) and signature ions (upper and lower branches) for the reaction of glutamine-spermidine adduct. The neutral loss of 71 Da from the initial adduct at 257 Da yields the observed signature ion at 186 Da. The observed signature ion at 212 Da is due to loss of CO and amine from the 257 Da adduct.

A second route for the formation of signature ions proceeds through ions formed by neutral loss. The upper pathway in Figure 10 presents a mechanism for the formation of a signature ion at 129 Da. This is created by a neutral loss of 71 Da from the initial 200 Da glutamate-putrescine adduct. This ion was observed 14 times. Subsequent loss of CO and amine does not create observed ions.  Figure 11 presents a similar mechanism for the formation of signature ions from the glutamine-spermidine adduct.

### 3.8. Neutral Loss Fragments from Polyaminated Adducts

**Figure 12:**
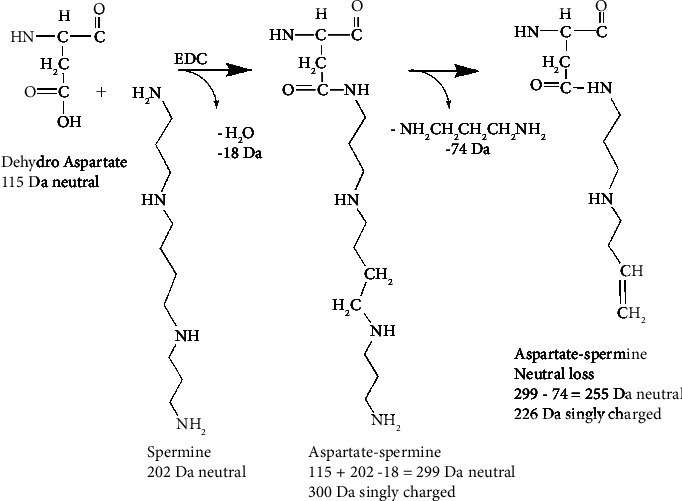
Scheme showing neutral loss of 74 Da from aspartate-spermine.

Occasionally, a portion of the polyamine is lost from the parent ion during the MS/MS fragmentation. This leaves a new parent ion of lower mass that can undergo subsequent fragmentation. Spermidine and putrescine adducts lose 71 Da, while spermine adducts lose 74 Da. Figure 10 shows a mechanism consistent with neutral loss of 71 Da from the glutamate-putrescine adduct.  Figure 11 shows a mechanism consistent with a neutral loss of 71 Da from glutamine-spermidine.  Figure 12 shows a mechanism consistent with a neutral loss of 74 Da from the aspartate-spermine adduct.

### 3.9. Manual Evaluation

Manual evaluation consists of identifying the sequence of amino acids in a peptide from the interval masses between peaks in an MS/MS spectrum. Most peaks will be singly charged, but doubly and triply charged peaks may also be present and must be sorted out. The intervals between the principal, singly charged peaks generally will be equal to the dehydro masses of amino acids, where a dehydro mass is the mass of the intact amino acid minus water. Lists of dehydro amino acid masses can be found in Lockridge and Schopfer 2022 supplement [10]. Care must be taken in locating the principal peaks because ancillary peaks due to loss of amine, water, and CO will also be present. Other studies have provided detailed strategies for the manual evaluation of MS/MS spectra [14].

**Figure 13:**
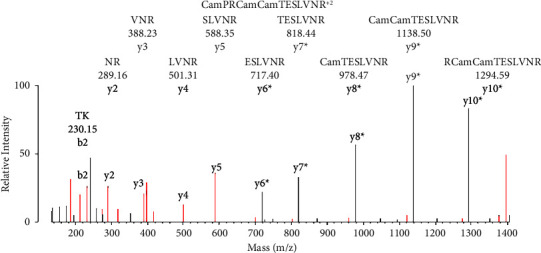
False positive spermine adduct on bovine serum albumin. Protein prospector assigned the sequence to TKCamCamTE(184.09)SLVNR with glutamate labeled by spermine and with carbamidomethylated cysteine residues (Cam). The parent ion mass in charge state +2 is 776.35 m/z. This assignment proved to be incorrect after manual evaluation. Fragments y2–y5 were assigned to the y-ion sequence SLVNR. The y6 fragment for Glu (184.09) expected to be at 901.48 Da was not found in the data. Rather, a mass at 717.40 Da was present that was consistent with an unlabeled glutamate. This fragment is labeled y6^*∗*^. The asterisk identifies the missed search engine assignment. Subsequent fragments y7^*∗*^–y10^*∗*^ are consistent with additional fragments that do not contain spermine. Fragments y6^*∗*^–y10^*∗*^ are not part of the Protein Prospector assignment.

The importance of manual evaluation for obtaining the full description of an MS/MS peptide fragmentation spectrum can be seen in Figures 3–7, where neutral loss and signature ions were revealed that would otherwise have gone unnoticed. Manual evaluation is also critical to identify incorrectly assigned candidates suggested by the search engine. An illustration of this latter role for manual evaluation can be found in Figure 13. The search engine assigned this MS/MS spectrum to peptide TKCamCamTE(184)SLVNR, where Cam = carbamidomethylated Cys, based on an N-terminal fragment consistent with TK, a y-ion sequence SLVNR, and the (M + 2H)^2+^ parent ion mass of 776.35 m/z. However, manual evaluation showed that the expected residue for the sixth position in the y-ion sequence, Glu 184, was not present. Rather, a strong fragment was present for the unlabeled glutamate residue at 717.39 Da. Subsequent fragments defined the sequence RCamCamTESLVNR with a fragment mass of 1294 Da for y10^*∗*^ and no label on glutamate. The BSA sequence in this region is TKCamCamTESLVNRRPCam for residues 498–600. The residue preceding the CamCam amino acid pair in the b-ion sequence is expected to be K. However, this is not what is observed. To explain this inconsistency, it is necessary to identify the residues at the N-terminus of this peptide. This can be done by examining the mass difference between y10^*∗*^ (1294 Da) and the singly charged parent ion (1551 Da). The difference between 1551 and 1294 is 257 Da which is consistent with P plus Cam. Thus, the observed sequence appears to be CamPRCamCamTESLVNR, with a singly charged parent ion mass of 1551 Da. However, CPR is not the N-terminal sequence for the CamCamTESLVNR peptide in bovine serum albumin. As such, it is not a sequence that would have been identified by the search engine. Manual evaluation was indispensable in assigning CamPRCamCamTESLVNR as the correct sequence for the peptide in Figure 13.

The question then becomes, how could the CamPRCamCamTESLVNR peptide appear in the tryptic digest of BSA since it is not in the primary sequence of BSA? The answer is protease-catalyzed rearrangement of amino acid residues. It has been observed that during tryptic digestion, a peptide containing a missed endo-proteinase cleavage site within 2-residues of either terminus can undergo a rearrangement in which the missed cleavage site from one terminus is transferred to the other terminus [15]. In our case, peptide CamCamTESLVNRRPCam with a missed cleavage site at RRPCam on the C-terminus can transfer the RPCam fragment to the N-terminus to create peptide CamPRCamCamTESLVNR. The mass of CamPRCamCamTESLVNR is equal to that of the [M+H]^+1^ parent ion, which is 1551 Da. The observed mass at 230 Da that Protein Pilot assigned as the N-terminal b2-ion, TK, is also consistent with CamP-28 or the a2-ion from the peptide CamPRCamCamTESLVNR.

## 4. Discussion

### 4.1. Methods for Detecting Polyaminated Proteins

The foundational studies by Folk and associates showed that the high abundance of polyamines inside cells could serve as transglutaminase substrates, promoting the catalytic activity of this enzyme and subsequent protein modification [16]. These investigations measured the incorporation of radioactive isotope-labeled putrescine into mitogen-stimulated human lymphocyte proteins. Mammalian transglutaminase requires calcium for activity, but the assay did not add calcium because it was known that mitogen stimulation causes an influx of calcium ions and increases endogenous transglutaminase activity. Three naturally occurring polyaminated proteins were detected by autoradiography on a 2D polyacrylamide gel. In a parallel study, the incorporation of radiolabeled ^14^C-putrescine was used by Song et al. to modify axonal tubulin [17].

The use of radioactive polyamines has been replaced by labeling with 5-(biotinamido) pentylamine for tracking polyaminated proteins. Biotinylated pentylamine labeling was introduced by Jeon et al. [18] and is currently the most popular reagent for detecting polyaminated proteins [19]. However, due to advancements in instrumentation and software, LC-MS/MS can now be used to detect both naturally occurring and laboratory-prepared polyaminated proteins [7, 20].

### 4.2. Limitations and Advantages of Methods That Identify Polyaminated Proteins

Polyamines are transported through the cell membrane, allowing intracellular proteins to be labeled in intact cells. Incorporation of radiolabeled polyamines, followed by SDS gel electrophoresis, identifies the number of polyaminated proteins in a specimen and their molecular weights. Immunoblotting with an antibody for the protein of interest can reveal whether the radioactive band and the antibody-bound band coincide. If they coincide, the evidence for the identity of the polyaminated protein is encouraging, but preliminary, because a band in an SDS gel can contain dozens of proteins migrating to the same position. Another weakness in this protocol is the need to have a good idea about the identity of the polyaminated protein so that the appropriate antibody can be used.

An advantage of using biotin pentylamine to label proteins is that the labeled proteins and peptides are easily enriched by binding to streptavidin beads. Western blotting with a streptavidin-labeled secondary antibody reveals the molecular weight of the polyaminated protein but not its identity. A disadvantage is the presence of endogenous biotinylated proteins, which are captured by streptavidin beads along with proteins labeled by biotin pentylamine. Labeling with biotin pentylamine does not identify the protein. The labeled proteins are identified by amino acid sequencing or by mass spectrometry of peptides released from streptavidin beads.

The advantage of mass spectrometry is that it identifies the polyaminated protein, the polyaminated peptide, the modified residue, and the polyamine. Mass spectrometry distinguishes between putrescine, spermidine, and spermine adducts. The major limitation of the mass spectrometry method is that the proteins of interest in a cell lysate must be enriched. Without enrichment or purification, finding polyaminated peptides is less likely. Enrichment strategies include immunoprecipitation, SDS gel electrophoresis, chromatography, and labeling with biotin pentylamine, followed by binding to streptavidin beads.

A mass spectrometry method called multiple reaction monitoring can find polyaminated peptides in a mixture of peptides if preliminary studies with the pure protein of interest have been completed. For example, in Figure 7, the model study with the purified recombinant protein identifies the mass of the modified peptide and the masses of fragment ions. This information allows the multiple reaction monitoring method to selectively search for parent ion mass 539.95 m/z and fragment ions 701.85, 804.41, and 941.47 m/z. The mass spectrometer ignores all other peptides in the cell lysate digest.

### 4.3. Naturally Occurring Polyaminated Proteins

The best known example for naturally occurring polyamination of proteins is the eukaryotic translation initiation factor eIF5A that is posttranslationally modified to incorporate a spermidine onto Lys 50. This modification is followed by hydroxylation of the spermidine adduct to make the amino acid hypusine [5]. Hypusinated eIF5A is essential for growth and survival of eukaryotic cells. A second example is the covalent attachment of putrescine or spermidine onto Gln 63 of RhoA, which leaves this GTPase in a constitutively active state [6]. Much less is known about polyamine adducts on bacterial proteins. One study reported that recombinant human histones expressed in *E. coli* are modified by putrescine, spermidine, and spermine via a covalent linkage to glutamine [7].

Bovine serum albumin binds polyamines noncovalently but there is no evidence for covalent modification [21].

## 5. Conclusion

Polyaminated adducts on proteins can be readily identified by mass spectral analysis. Identification is enhanced by the presence of signature ions. Manual evaluation is an indispensable factor in the convincing identification of polyaminated peptide adducts.

## Figures and Tables

**Table 1:** Neutral masses of polyamine adducts on glutamate, glutamine, and aspartate.

Polyamine	Amino acid residue	Catalyst	Dehydro residue	Polyamine added mass	Adduct neutral mass
Putrescine	Glutamate	EDC	129.04	70.06	199.10
Putrescine	Glutamine	Transglutaminase	128.05	71.07	199.12
Spermidine	Glutamate	EDC	129.04	127.12	256.16
Spermidine	Glutamine	Transglutaminase	128.05	128.13	256.18
Spermine	Glutamate	EDC	129.04	184.18	313.22
Spermine	Glutamine	Transglutaminase	128.05	185.19	313.24
Putrescine	Aspartate	EDC	115.02	70.06	185.08
Spermidine	Aspartate	EDC	115.02	127.12	242.14
Spermine	Aspartate	EDC	115.02	184.18	299.20

There are no entries in Table 1 for transglutaminase adducts on glutamate or aspartate because transglutaminase only labels glutamine.

**Table 2:** Polyamine modified peptides in bovine serum albumin that are labeled by the action of EDC on glutamate and aspartate.

Polyaminated BSA peptide	Labeled residue	Polyamine	Signature ions Da	Neutral loss, Da	Discriminant score
NE^*∗*^LTEFAK	E69	Spermidine		71	4.27
NE^*∗*^LTEFAK	E69	Putrescine	155, 200		5.31
VNE^*∗*^LTEFAK	E69	Putrescine	155, 200		5.52
LVNE^*∗*^LTEF	E69	Putrescine	155		6.35
LVNE^*∗*^LTEFAK	E69	Putrescine	155, 200		5.16
LVNELTE^*∗*^FAK	E72	Putrescine	129, 155, 200		3.89
SLHTLFGDE^*∗*^LCK	E97	Putrescine	129		5.95
NE^*∗*^CFLSKK	E124	Spermine	195, 269		3.86
LKPDPNTLCDE^*∗*^FK	E149	Spermidine			6.41
YNGVFQECCQAE^*∗*^DK	E195	Putrescine			4.59
YYANKYNGVFQECCQAE^*∗*^DK	E195	Putrescine			6.34
HPYFYAPELLYYANKYNGVFQECCQAE^*∗*^DK	E195	Putrescine			7.20
AE^*∗*^FVEVTKLVTDLTK	E250	Putrescine	155, 200		6.68
LSQKFPKAE^*∗*^FVEVTK	E250	Putrescine			6.03
LVTDLTKVHKECCHGDLLE^*∗*^CADDR	E275	Putrescine			7.34
DAIPE^*∗*^NLPPLTADFAEDK	E323	Putrescine			6.58
DAIPENLPPLTADFAE^*∗*^DKDVCK	E334	Putrescine			7.64
DAIPENLPPLTADFAE^*∗*^DKDVCK	E334	Spermidine	186		6.61
PENLPPLTADFAE^*∗*^DKDVCK	E334	Putrescine			8.15
NYQE^*∗*^AKDAFLGSFLYEYSR	E344	Putrescine			6.89
DVCKNYQE^*∗*^AKDAFLGSFLYEYSR	E344	Putrescine			8.55
LGSFLYE^*∗*^Y	E356	Putrescine	129, 155, 200		3.89
DAFLGSFLYE^*∗*^Y	E356	Putrescine	155, 200		5.30
DAFLGSFLYE^*∗*^Y	E356	Spermine	269	74	4.30
DAFLGSFLYE^*∗*^Y	E356	Spermidine			4.12
DAFLGSFLYE^*∗*^YSR	E356	Putrescine			6.64
DAFLGSFLYE^*∗*^YSR	E356	Spermidine	186	71	4.98
HPE^*∗*^YAVSVLLR	E363	Putrescine	129		5.88
HLVDE^*∗*^PQNLIK	E406	Putrescine	155		4.67
LGE^*∗*^YGFQNALIVR	E423	Putrescine	155		6.40
LGE^*∗*^YGFQNALIVR	E423	Spermine	195, 269		4.97
LGE^*∗*^YGFQNALIVR	E423	Spermine	195, 269		4.86
PTLVE^*∗*^VSR	E448	Putrescine	129		5.74
PTLVE^*∗*^VSR	E448	Spermidine		71	3.81
TPTLVE^*∗*^VSR	E448	Putrescine	129		6.51
TPTLVE^*∗*^VSR	E448	Spermidine		71	4.36
STPTLVE^*∗*^VSR	E448	Putrescine	129		5.67
VSTPTLVE^*∗*^VSR	E448	Putrescine	129		6.73
VPQVSTPTLVE^*∗*^VS	E448	Spermine	269		4.67
VPQVSTPTLVE^*∗*^VSR	E448	Putrescine			7.75
VPQVSTPTLVE^*∗*^VSR	E448	Spermidine		71	6.88
KVPQVSTPTLVE^*∗*^VSR	E448	Putrescine			7.62
KVPQVSTPTLVE^*∗*^V	E448	Spermidine			4.85
YTRKVPQVSTPTLVE^*∗*^VSR	E448	Putrescine			7.20
TE^*∗*^DYLSLILNR	E473	Putrescine			6.52
TVME^*∗*^NGVAFVDK	E512	Spermine	195, 269	74	4.49
TVME^*∗*^NGVAFVDK	E512	Putrescine	200		5.20
PDE^*∗*^TYVPK	E518	Putrescine	155		5.58
PDE^*∗*^TYVPK	E518	Spermidine			3.54
RPCFSALTPDE^*∗*^TYVPK	E518	Putrescine	155		5.32
LFTFHADICTLPDTE^*∗*^K	E543	Putrescine			6.50
SLHTLFGD^*∗*^ELCK	D96	Putrescine	141		6.03
PNTLCD^*∗*^EFK	D48	Putrescine	141		4.41
NLPPLTAD^*∗*^FAEDK	D331	Putrescine	141		6.58
PENLPPLTAD^*∗*^FAEDK	D331	Putrescine	141		7.39
D^*∗*^AIPENLPPLTADFAEDK	D319	Spermine	300		6.13
DAIPENLPPLTAD^*∗*^F	D331	Spermine	272	74	5.31
DAIPENLPPLTAD^*∗*^F	D331	Putrescine			6.41
DAIPENLPPLTADFAED^*∗*^K	D335	Spermidine			6.77
D^*∗*^AFLGSFLYEY	D347	Putrescine			4.09
D^*∗*^AFLGSFLYEYSR	D347	Spermine			7.01
D^*∗*^AFLGSFLYEYSR	D347	Spermine			4.96
D^*∗*^AFLGSFLYEYSR	D347	Spermine			7.10
D^*∗*^AFLGSFLYEYSR	D347	Spermine	226		5.25
D^*∗*^AFLGSFLYEYSR	D347	Putrescine	186		6.01
D^*∗*^AFLGSFLYEYSR	D347	Spermidine	198		5.92
TED^*∗*^YLSLILNR	D474	Spermine	272		5.97
TED^*∗*^YLSLILNR	D474	Putrescine			6.14
PCTED^*∗*^YLSLILNR	D474	Putrescine			8.61
MPCTED^*∗*^YLSLILNR	D474	Putrescine	186		7.32
LFTFHAD^*∗*^ICTLPDTEK	D535	Putrescine			7.55
TFHADICTLPD^*∗*^TEK	D541	Putrescine			4.88
LFTFHADICTLPD^*∗*^TEK	D541	Spermidine			6.57
TVMENGVAFVD^*∗*^	D579	Putrescine	186		5.11
TVMENGVAFVD^*∗*^K	D579	Spermine		74	3.89
TVMENGVAFVD^*∗*^K	D579	Spermine			4.25
TVMENGVAFVD^*∗*^K	D579	Spermine	226	74	4.47
TVMENGVAFVD^*∗*^K	D579	Putrescine	186		5.46
TVMoxENGVAFVD^*∗*^K	D579	Spermine		74	3.70
TVMENGVAFVD^*∗*^KCCADDK	D579	Putrescine			5.69

Cysteines are carbamidomethylated, adding 57 Da to the mass of the peptide. A discriminant score greater than 0.0 is considered to be strong evidence for a correct match of the peptide sequence to the MS/MS spectrum [11].

**Table 3:** Polyamine modified peptides in rUsp/His_6_ that are labeled by the action of microbial transglutaminase.

Polyaminated rUsp/His_6_ peptide	Labeled residue	Polyamine	Signature ions, Da	Discriminant score
IVDFQ^*∗*^HSIEQEAK	Q55	Putrescine	155	5.79
PSIVDFQ^*∗*^HSIEQEAK	Q55	Spermidine		9.07
APSIVDFQ^*∗*^HSIEQEAK	Q55	Putrescine	200	8.38
APSIVDFQ^*∗*^HSIEQEAK	Q55	Spermidine	212	8.66
APFAPSIVDFQ^*∗*^HSIEQEAK	Q55	Putrescine	200	8.42
APSIVDFQHSIEQ^*∗*^EAK	Q60	Spermine		4.36
APSIVDFQHSIEQ^*∗*^EAK	Q60	Spermidine		6.10
PFAPSIVDFQHSIEQ^*∗*^EAK	Q60	Spermidine		7.25
PFAPSIVDFQHSIEQ^*∗*^EAK	Q60	Spermine		6.34
Q^*∗*^ANVDVLVVR	Q267	Spermine		5.58
Q^*∗*^ANVDVLVVR	Q267	Putrescine	129	6.34
SILHQ^*∗*^ANVDVLVVR	Q267	Putrescine		6.96
FFLGSTANSILHQ^*∗*^ANVDVLVVR	Q267	Putrescine		11.60
FFLGSTANSILHQ^*∗*^ANVDVLVVR	Q267	Spermine		6.25
FFLGSTANSILHQ^*∗*^ANVDVLVVR	Q267	Spermidine	186	8.29

A discriminant score greater than 0.0 is considered to be strong evidence for a correct match of the peptide sequence to the MS/MS spectrum [11].

**Table 4:** Putrescine modified peptides in bovine serum albumin that are labeled by the action of microbial transglutaminase.

BSA peptide	Labeled residue	Polyamine	Signature ions, Da	Discriminant score
GLVLIAFSQ^*∗*^Y	Q53	Putrescine		4.75
IAFSQYLQQ^*∗*^CPFDEHVK	Q57	Putrescine		6.97
LIAFSQYLQQ^*∗*^CPFDEHVK	Q57	Putrescine		6.52
GLVLIAFSQYLQQ^*∗*^CPFDEH	Q57	Putrescine		6.90
GLVLIAFSQYLQQ^*∗*^CPFDEHVKVK	Q57	Putrescine		4.41
YNGVFQECCQ^*∗*^AEDK	Q193	Putrescine		6.45
YICDNQ^*∗*^DTISSK	Q291	Putrescine	155	6.57
PENLPPLTADFAEDKDVCKNYQ^*∗*^EAK	Q343	Putrescine		8.63
HLVDEPQ^*∗*^NLIK	Q408	Putrescine		5.79
HLVDEPQNLIKQ^*∗*^NCDQFEK	Q413	Putrescine		9.01
LGEYGFQ^*∗*^NAL	Q427	Putrescine	200	5.20
GEYGFQ^*∗*^NALIVR	Q427	Putrescine	129, 155, 200	6.52
LGEYGFQ^*∗*^NALIVR	Q427	Putrescine	155, 200	8.21
VPQ^*∗*^VSTPTLVEVSR	Q440	Putrescine		6.64
PDTEKQ^*∗*^IK	Q545	Putrescine	155, 200	6.06
TLPDTEKQ^*∗*^IK	Q545	Putrescine		5.63
TFHADICTLPDTEKQ^*∗*^IK	Q545	Putrescine		5.85
KQ^*∗*^TALVELLK	Q549	Putrescine	155	4.68
LVVSTQ^*∗*^TAL	Q603	Putrescine	155	5.58

Cysteines are carbamidomethylated, adding 57 Da to the mass of the peptide. A discriminant score greater than 0.0 is considered to be strong evidence for a correct match of the peptide sequence to the MS/MS spectrum [11].

**Table 5:** Signature ions for polyamine adducts on glutamate, glutamine, and aspartate.

	Glutamate	Glutamine	Aspartate
Putrescine	**200 (8)**, 172, **155 (10)** **129 (8)**, 101, 84	**200 (14)**, 172, **155 (7)** **129 (2)**, 101, 84	**186 (5)**, 158, **141 (4)** 115, 67, 50

Spermidine	257, 229, 212 **186 (2)**, 158, 141	257, 229, **212 (1)** **186 (1)**, 158, 141	243, 215, **198 (1)** 172 144, 127

Spermine	314, 286, **269 (6)** 240, 212, **195 (4)**	314, 286, 269 240, 212, 195	**300 (1)**, **272 (2)**, 255 **226 (2)**, 198, 181

Observed ions are shown in bold font, while theoretical ions are shown in black. Numbers in brackets indicate the number of times the signature ion was observed.

## Data Availability

The mass spectrometry data have been deposited to the ProteomeXchange Consortium via the PRIDE partner repository (Perez-Riverol et al. 2022. Nucleic Acids Res 50 (D1): D543-D552) with the dataset identifier PCD046396.

## Abbreviations

LC-MS/MS: Liquid chromatography tandem mass spectrometry
EDC: 1-Ethyl-3-(3-dimethylaminopropyl) carbodiimide
BSA: Bovine serum albumin
Usp: *Francisella tularensis* universal stress protein
rUsp/His_6_: Recombinant universal stress protein with a C-terminal histidine tag.
